# Prime editing: therapeutic advances and mechanistic insights

**DOI:** 10.1038/s41434-024-00499-1

**Published:** 2024-11-28

**Authors:** Joss B. Murray, Patrick T. Harrison, Janine Scholefield

**Affiliations:** 1 Department of Physiology, University College Cork, Cork, Ireland; 2 Division of Pulmonary Medicine, Cincinnati Children’s Hospital, Cincinnati, OH USA; 3 Bioengineering and Integrated Genomics, NextGen Health, CSIR, Pretoria, South Africa

**Keywords:** Genetic engineering, Diseases

## Abstract

We are often confronted with a simple question, “which gene editing technique is the best?”; the simple answer is “there isn’t one”. In 2021, a year after prime editing first made its mark, we evaluated the landscape of this potentially transformative advance in genome engineering towards getting treatments to the clinic [1]. Nearly 20% of the papers we cited were still in pre-print at the time which serves to indicate how early-stage the knowledge base was at that time. Now, three years later, we take a look at the landscape and ask what has been learnt to ensure this tech is broadly accessible, highlighting some key advances, especially those that push this towards the clinic. A big part of the appeal of prime editing is its ability to precisely edit DNA without double stranded breaks, and to install any of the 12 possible single-nucleotide conversion events as well as small insertions and/or deletions, or essentially any combination thereof. Over the last few decades, other transformative and Nobel prize-winning technologies that rely on Watson-Crick base-pairing such as PCR, site-directed mutagenesis, RNA interference, and one might say, “classic” CRISPR, were swiftly adopted across labs around the world because of the speed with which mechanistic rules governing their efficiency were determined. Whilst this perspective focuses on the context of gene therapy applications of prime editing, we also further look at the recent studies which have increased our understanding of the mechanism of PEs and simultaneously improved the efficiency and diversity of the PE toolbox.

## Introduction

### Overview of prime editing mechanism

**Fig. 1:**
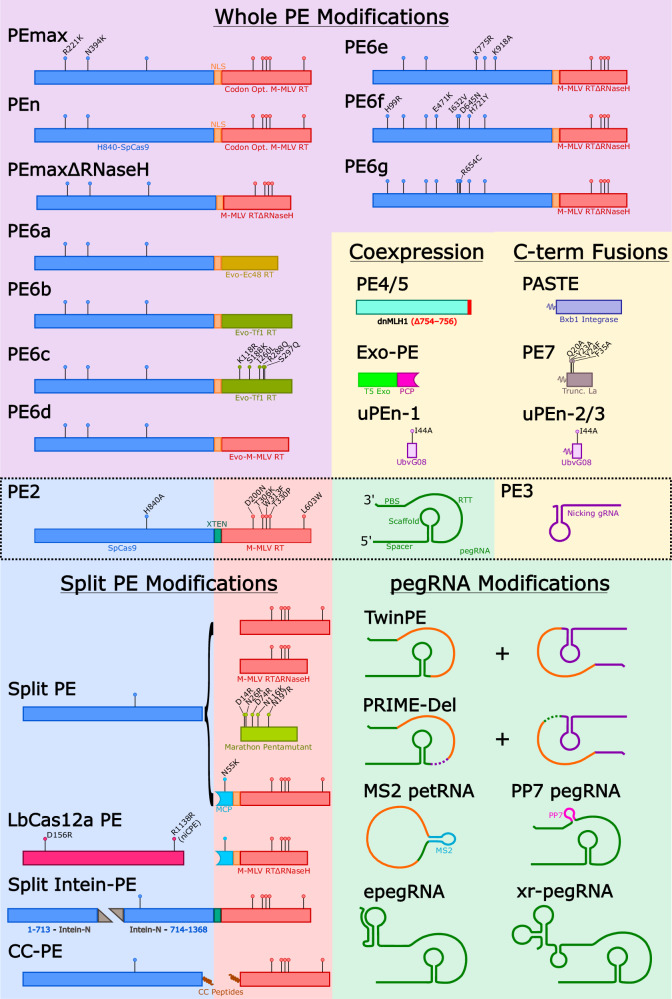
Structural schema of current PE technologies.

The original and most widely used to date Prime Editor (PE) protein is PE2 (see Fig. 1), a fusion of the *S. pyogenes* Cas9 nickase (with its HNH domain inactivated by the H840A amino acid substitution) and an engineered pentamutant version of Mouse-Moloney Leukaemia Virus reverse transcriptase (M-MLV RT with five amino acid substitutions: D200N/T306K/W313F/T330P/L603P) [2]. To initiate editing, the Cas9 nickase portion of PE2 binds to a short DNA sequence known as the protospacer adjacent motif (PAM; typically the 3-nt canonical sequence, NGG), and is then guided to its specific genomic DNA target site by an elongated prime editing guide RNA (pegRNA) whereupon it nicks the genomic DNA 3-nt upstream of the PAM; this liberates two stretches of single stranded DNA known as the 3’ flap and 5’ flap. The 3’ flap from the genomic DNA hybridises to a customised sequence at the 3’ end of the pegRNA known as the primer binding site (PBS), and this serves to prime the reverse transcription process. Once initiated, the reverse transcriptase (RT) enzyme then extends the 3’ DNA flap sequence using the RT template (RTT) sequence (alternatively referred to as the edit sequence) which is also included in the pegRNA design. It is postulated that the extended 3’ flap (which now contains the desired edit) can displace the unmodified 5’ flap to create a mismatched double-stranded DNA sequence with the desired edit on one strand, and the original sequence on the other. If resolved by mismatch repair (MMR) in favour of the edited strand, this completes the editing in genomic DNA. It should be noted that this original method of prime editing was called PE2, which is also the name given to Cas9 H840A nickase-RT fusion protein in several of the subsequent improved versions of prime editing. For example, the PE3 strategy of prime editing which uses the PE2 fusion protein with a pegRNA as well as coadministration of a so-called nicking guide RNA (ngRNA), which targets the non-edited strand at a suitable distance from the edit, can boost editing efficiency by 2- to 4-fold [2, 3]. It was postulated that addition of this ngRNA further biases DNA repair towards the edited strand, thereby improving intended editing efficiency, a strategy originally introduced with base editing [4]. This strategy of using the PE2 editor (Cas9 nickase-RT fusion protein) with the nicking guide is referred to simply as PE3 [2].

### Advantages and applications of Prime Editing

The two main advantages of prime editing over previous techniques lies in the ability to a) create specific changes to the DNA without the use of double stranded breaks (DSB) thus substantially reducing the incidence and consequences of off-target effects, and b) introduce a range of precise changes in both dividing *and* non-dividing cells without bystander edits. By way of example, both benefits were seen in an early study comparing prime editing, base editing and homology directed repair (HDR) to correct the cystic fibrosis (CF) causing variant R785X [5]. This study also highlighted the relatively low efficiency of the original prime editing strategy relative to adenine base editing (ABE) which could also be used to correct this particular mutation.

**Fig. 2:**
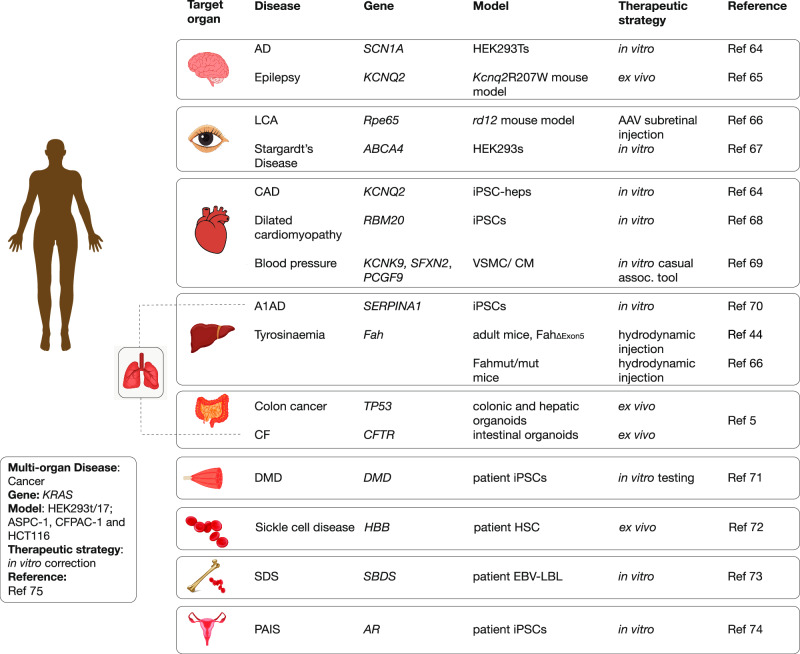
Highlights of prime editing research applications in terms of diseases.

The repertoire of potential prime edits includes all possible single nucleotide changes, as well as correction of both small insertions and deletions. Indeed, analyses of ClinVar data indicates as many as 16,000 small deletions could be repaired using prime editing for therapeutic purposes [6]. As a research tool, PE enables a myriad of creative modifications such as targeted in-frame insertions of short peptide tags to modify and/or track a protein’s location, and structural changes to DNA regulatory regions. As discussed later, the PE strategy can also insert a DNA sequence to act as a “landing pad” for incorporation of much larger DNA sequences via the use of recombinases. Several reviews have covered the numerous publications that are showcasing the diverse disease applications of prime editing [7, 8], and we highlighted some of these in terms of disease areas in Fig. 2. This encouragingly shows the breadth of clinical applications by disease organ class that are being investigated using prime editing approaches.

## Designer PEs

The next generation of PEs after PE2 was developed with the goal of improving editing efficiency. The implementation of the mutations R221K and N394K in the Cas9 region of PE2, in combination with codon optimisation of the RT and replacement of the linker region with a nuclear localisation sequence was found to improve editing efficiency compared to the parental PE2 [9]. This modified PE was subsequently designated PEmax due to its superior editing efficiency. It was later shown that truncation of the RNaseH domain of the PEmax RT did not hinder editing efficiency [10] thereby introducing a highly efficient and comparably smaller PE named PEmaxΔRNaseH.

### Evolved PEs

David Liu and colleagues have added to the suite of smaller PEs to overcome the challenge of cargo size. However, having initially tried screening a large range of naturally occurring RTs from diverse phylogenetic origins, as well as a rational engineering approach to create improved PEs without improvements on the original, the authors switched to a phage-assisted continuous evolution (PACE) approach [11]. One aim was to make a more compact PE to enhance delivery options using two different targets, either a 1 bp insertion (v1) or a 20 bp insertion (v2; referred to as v3 when used with an epegRNA targeting the opposite strand).

Rather than identifying a single optimised PE2 with the lab-based evolution approach, the authors observed *two* different PE2 variants which had specialised editing ability depending on the editing target. Specifically, the PE evolved for the +1 insertion (v1) worked ~10-fold better than the starting PE2 but was ~10-fold worse at the +20 edit (v2). In contrast, the PE evolved for a + 20 insert was ~50 fold better than the starting PE but was ~10 fold worse at the +1 edit. The evolution of highly processive PEs capable of complex editing may explain this, where editors with higher than necessary activity for the edit, risk integration of the pegRNA scaffold leading to weak selection during evolution. Alternatively, editors with limited activity will not fully process the RTT. These observations underline our opening response to the question “which gene editing technique is the best?”; for prime editing, the answer is very much, “it depends…”.

To further investigate this, the study subjected a compact RT from *E. coli* (Ec48) to multiple passages on a second lab-based evolution process known as v1 and v2 PANCE (phage-assisted non-continuous evolution), with the outcome of an evolved Ec48. Three of the mutations identified in this process (E60K, E279K, and K318E) localised in the vicinity of the Ec48 DNA-RNA substrate, suggesting they may improve the interaction between the RT and the DNA-RNA complex. This evolved Ec48 is the RT component of PE6a, which offered greater editing efficiency compared to a PE composed of a pentamutant Marathon RT with comparable size. It must be noted that editing efficiencies see marked improvements when PE6a is combined with pegRNAs modified with a 3’ pseudoknot motif.

Evolving another compact RT, Tf1 from *S. pombe*, using multiple passages of v1, v2 and v3 PANCE produced an evolved Tf1 with improved efficiency compared to WT [11]. As a PE, the evolved Tf1 is the RT component of PE6b which was considerably smaller than PEmax yet retained comparable editing efficiency. PE6b was able to correct the 1278insTATC mutation in *HEXA* in Tay-Sachs patient fibroblasts at a higher editing efficiency than PEmax whilst ~33% smaller in cargo size (PE6a = 1.2 kb, PE6b = 1.5 kb, PEmax = 2.2 kb).

To create PE6c, the Liu lab included five mutations from their rational design study [11] proximal to the DNA-RNA substrate domain. This led to greater processivity, making a strong candidate for edits requiring pegRNAs with complex RTTs and prime editing systems that require longer, distal pegRNAs such as twinPE. For example, twinPE using PE6c was able to insert a 38 bp *attB* site into on average 34% of T cells compared to 22% using PEmax.

To improve the editing efficiency of the RT domain of PE2, M-MLV was evolved in three parallel evolution circuits followed by collection and screening of the subsequent mutations [11]. It was evident that complete deletion of the RNaseH domain, as is the case in PEmaxΔRNaseH, may not be required to optimise editing as a premature termination near the beginning of the RNaseH domain arose from the PE2 evolution campaign. Additional mutations proximal to the polymerase active site were combined with this RNaseH truncation to create the PE PE6d. Prime editing using PE6d excelled at installing edits from RTTs with complex structural elements such as *loxP* insertion RTT which has a predicted 13-nt hairpin structure. For this edit, 30% of *loxP* inserts were prematurely truncated using PEmaxΔRNaseH, compared to only 5.8% using PE6d. A side effect of the high processivity of PE6d, however, is an increased rate of pegRNA scaffold insertion. Therefore, the application of PE6d is not advised in the case of simple prime edits where the scaffold is more likely to be integrated. In a split intein system across three AAVs, PE6d was compared to PEmaxΔRNaseH for the ability to insert a *loxP* site into the *Dnmt1* locus of C57BL/6 mice by twinPE. Three weeks after intracerebral injection into neonatal mice, PE6d was able to install the *loxP* site into 40% of cells harvested from murine cortices, compared to just 1.7% with the PEmaxΔRNaseH system. This illustrates the potency of these evolved RTs in an in vivo AAV delivery system compared to the preceding generation.

Further evolution campaigns were applied to the whole PE2 to identify Cas9 mutations that will benefit prime editing. After consolidating the beneficial mutations in different combinations, further PEs were produced with the designations PE6e/f/g. At multiple loci with different edit types, these new PEs offered nearly double the editing efficiency compared to PEmaxΔRNaseH. It was emphasised that different edits may require a specific PE for optimal efficiencies, and to address this the evolved Cas9 of PE6e/f/g was combined with the evolved RT of PE6a/b/c/d to tailor prime editing to the edit type. For example, the smaller PE6a exhibited reduced editing of *CXCR4* and *IL2RB* compared to PEmax. However, when the evolved Ec48 RT of PE6a was combined with the evolved Cas9 of PE6e, the resulting PE6a/e editor accomplishes editing efficiencies comparable to PEmax with a fraction of the size. The modular nature of this new collection of PEs demonstrates that optimal editing efficiency may require customising the PE as well as the pegRNA. Furthermore, these smaller and potent PEs compete with the efficacy of the previous generation, while also facilitating viral delivery systems.

### Nuclease-based Prime Editing

Another consideration for prime editing efficiency concerns the decision point between accepting the edit that extends the 3’ flap by reverse transcription or maintaining the unedited sequence on the 5’ flap. Peterka and colleagues [12] address this by employing a fully active Cas9 nuclease in a PE system rather than a H840A nickase. This system installs edits after a DSB, leading to a blunt end rather than a 5’ flap. To resolve the potential for DSB-related indels, the 5’ end of the pegRNA was designed to be reverse transcribed into a sequence homologous to a region downstream of the DSB. Using this strategy, PE nuclease (PEn) was able to install insertions at efficiencies comparable to or better than PE2 at many loci, with the caveat of increased imprecise edits and indels. It was proposed that the precise edits utilised the 5’ homology tail of the pegRNA to mimic HDR and insert the edit, while many imprecise edits including the insertion of the homology tail may be mediated by non-homologous end joining (NHEJ) to resolve the DSB. Indeed, treatment with AZD7648, an inhibitor of the NHEJ repair protein DNA-PK [13], eliminated most imprecise edits using PEn and enhanced precise editing to above PE2 levels at many loci. In agreement, PEn editing using a pegRNA with no homology tail co-treated with AZD7648 dramatically reduced editing efficiency. In further work, simultaneous inhibition of DNA-PK and Polϴ also reduced indels [14], illustrating the influence of NHEJ on PEn. Li and colleagues [15] explored an alternative enhancement of PEn via inhibition of 53BP1-mediated NHEJ. Overexpression of a ubiquitin variant exploits the 53BP1 recognition of histone 2A K15 ubiquitination at DSBs, leading to 53BP1 inhibition and skewing DNA repair mechanisms towards HDR rather than NHEJ [16]. Delivery of PEn, with this ubiquitin variant conjugated to the RT was designated ubiquitin variant-assisted PEn (uPEn), and exhibited enhanced editing efficiencies for insertion, deletion and replacement edits at multiple loci compared to PEmax, PE3max and PE5max. These uPEn systems greatly benefit from a homology tail in the pegRNA to further bias repair away from NHEJ, but despite this uPEn suffers more unintended edits, likely due to DSB-related indels.

## Split PEs

Given that even the basic version of PE2 is encoded by >6 kb of sequence, adoption of this method into the most commonly applied gene therapy vehicle pipelines, (i.e. AAV with a cargo capacity of only ~5 kb) will require a number of modifications to the existing PE architecture. One approach, used by both Joung and colleagues [17], and Sontheimer and colleagues [18] was to simply uncouple the reverse transcriptase (RT) from the Cas9 nickase, which resulted in similar frequencies of editing across multiple target sites in multiple cell lines. In addition to facilitating delivery options, the observation that the untethered RT is capable of catalysing the extension step indicates that this may also be the case with Cas9-RT fusion proteins i.e. the Cas9 domain of one molecule of PE binds and nicks the DNA, with the RT domain of a second non-DNA bound PE molecule available to reverse transcribe the RTT. This also raises the theoretical possibility of a PE molecule extending an off-target genomic site where a nicked DNA/RNA hybrid might exist [17].

To improve the efficacy of these split PE systems, the phage M coat protein (MCP) system has been applied, which recognises MS2 RNA aptamers. The inclusion of an MS2 aptamer sequence in the pegRNA facilitates recognition by an M coat protein (MCP) fused RT. The MCP-RT is split from the Cas9 allowing for the latter construct to be loaded into a separate AAV from the remaining components [18]. In addition, the RTT-PBS portion of the pegRNA including the MS2 aptamer, termed the prime editing template RNA (petRNA) is split from the spacer and scaffold, and circularised using flanking ribozyme sequences. Circularising the petRNA also improved stability and integrity, with concomitant editing efficiencies rivalling that of a traditional pegRNA for a 3-nt substitution in the *FANCF* locus. Another strategy for recruiting a split RT to a Cas for prime editing employs coiled-coil peptides fused to the termini of each protein. This coiled-coil prime editing (CC-PE) system allows substitution of PAM-flexible SpCas9 modules such as SpRY, emphasising customisability and delivery using dual AAVs [19].

Another advantage to these split PE systems is the propensity to combine different PE components without engineering their fusion. The bacterial group II intronic reverse transcriptase Marathon [17] was investigated as an alternative to M-MLV-RT due to its smaller size. Five rationally designed mutations improved the editing efficiency of PE systems using Marathon, further enhanced by untethering the pentamutant Marathon from the nickase. Marathon-RT is 753 bp smaller than the original M-MLV RT, however editing efficiency is sacrificed for this size improvement. Besides the RT, the Cas portion of PE systems can also be easily substituted with a split system, as was shown with SaCas9 [17] and LbCas12a [20]. These split PE systems introduce a novel customisability as well as deliverability aspect to the prime editing field which will require further innovation.

Other attempts to reduce the cargo size have focused on a truncated version of the RT ( > 25% shorter than the full length) such as the Mini-PE which was created by screening for the smallest functional M-MLV RT fused to *C. jejuni* Cas9 nickase (H559A) [21] and showed reliable functionality. The advantage of Mini-PE compared to other PE systems comes in its reduced size of 4.5 kb, which is amenable to AAV packaging. Delivery of Mini-PE by AAV with a separate AAV prepared carrying the pegRNA and ngRNA into mouse retinas revealed low but detectable editing of *Hsf1*. While efficiency was low (0.6%), it was promising to observe prime editing following delivery of these components via AAV to the mouse retina, encouragingly showing potential value in the clinic.

## pegRNA modifications

The highly modular nature of PEs is a double-edged sword by allowing for control over the optimal design of each portion, but at the same time, requiring high-throughput modelling to predict how the many variable components work together in concert to enact efficient target editing. Our previous perspective highlighted the extensive work now published [22], which established predictive design strategies after screening over 50,000 pegRNAs using a lentiviral library. Despite using an exogenous targeting approach, important papers such as these, set the foundation for how important AI would be in predicting efficiency of prime editing. Considering the immense scope for pegRNA optimisation required for identifying the most efficient prime editing system for the desired edit, a novel strategy for pegRNA construction was developed to produce pegRNA libraries [23]. This Multiplexing Of Site-specific Alterations for In situ Characterization (MOSAIC) method allows for the production of linear cassettes encoding a plethora of pegRNAs that install 4-nt insertion barcodes into specified loci. Identification of the most efficiently installed insertion will pinpoint the ideal PBS and RTT length for prime editing of that locus, thereby streamlining an arduous component of the prime editing workflow. This MOSAIC pegRNA library method was applied to identify optimal pegRNAs for the installation of transcription factor consensus sequences with the aim of exploiting endogenous transcription factors for durable gene expression alterations [24].

### pegRNA design tools

Despite the initial focus on all manner of single nucleotide changes made possible by prime editing, the same methodology has been increasingly used for these small insertions and deletions, for which the importance has been outlined earlier. Using lentiviral libraries containing target sites against multiple genes in two different cell lines, Koeppel and colleagues [25] assessed the insertion efficiencies of over 3500 pegRNAs of insertions varying from 1–69 nucleotides. This in-depth study revealed several points of interest, firstly, noting that insertion rates were more closely correlated between the same target sites across HEK293T and HAP1 cells than between different target sites within the same cell line. As expected, MMR status was identified as a primary cause of variation between tested lines (within the context of shorter sequences). Other predictors including the inhibitory effects of TREX1/2 over-expression, in a length-dependent manner, were supported by observations of the protective effects of pegRNA secondary structure, again within the context of longer sequences (of note, others have shown that TREX1 expression can also impact HDR editing [26]). Koeppel and colleagues [25] also observed a strong preference for cytosines, which revealed an opportunity in the context of selecting optimal codons for useful tags such as His6. Finally, whittling down the multiple contributing factors to 10 features, they have created a publicly available insertion efficiency prediction model, MinsePIE [25], with an average predictive power of 0.68.

Another recent deep-learning publication of interest combined self-targeting lentiviral libraries to develop PRIDICT, which the authors indicate reaches high accuracy across single nucleotide replacements, indels, and unintended editing at the target [27]. Using a library of 119,701 pegRNAs targeting 14,238 pathogenic mutations, and after filtering for various features, their machine learning model, revealed predictions with a correlation of R = 0.85/r = 0.61. Importantly, the study revealed lower but predictable PE in vivo in mouse hepatocytes. Here, setting a PRIDICT threshold of 70, led to a ~ 6 fold (tevopreQ1-modified epegRNAs) and ~10 fold (unmodified pegRNAs) increase in median PE efficiency which justifies the value-add of using prediction models to get closer to the clinic. Further developments to the PRIDICT system gave rise to PRIDICT2.0 which estimates efficiency of prime edits of 1–5 bp replacements or 1–15 bp insertions/deletions [28]. Another widely used tool that also uses machine learning for design optimisation of pegRNAs, OPED (Optimised Prime Editing Design), was validated using a broad range of datasets. The developers have created an OPEDVar database of refined pegRNA designs for >2 billion candidates for all reported pathogenic variants [29]. Similarly, the DeepPrime prediction tool builds from the previous DeepPE, and is trained on the editing efficiencies of 338,996 pegRNAs and 3979 epegRNAs, adding to the prime editing predictive tools available for use [30]. However, it must be emphasised that while these tools may be valuable for pegRNA design, this does not replace experimental testing. In support of this, a custom designed and rigorously tested epegRNA for the correction of the F508del mutation in the *CFTR* gene exhibited superior editing efficiency when compared to the top suggested epegRNAs from both PRIDICT and DeepPrime [31].

### pegRNA stabilisers

The secondary structure of RNA duplexes can impact prime editing efficiency. In creating a 3’ trimmed evopreQ1 (tevopreQ1) or mpknot RNA pseudoknot to stabilise plasmid-expressed pegRNAs, David Liu’s team provided a mechanism for limiting 3’ exonuclease degradation of the pegRNA [32]. Addition of these 3’ motifs to a pegRNA is denoted as an engineered pegRNA (epegRNA). In some cases, a linker sequence between the PBS and 3’ motif was shown to further improve editing efficiency; however, this too requires careful design. Helpfully, these linker regions can be designed to limit complementarity with the PBS or spacer using an openly accessible design tool based on ViennaRNA [33] known as pegLIT (https://peglit.liugroup.us). The hypothesised stability improvement of these epegRNAs indeed resulted in improved editing efficiency, introducing a relatively easy refinement. In support of this, the simple addition of a tevopreQ1 pseudoknot to a pegRNA containing a 13-nt PBS and RTT of 10-nt homology beyond the target edit with an 8-nt linker consistently surpassed the prime editing efficiency of the comparable unmodified pegRNA for nine different loci. A slightly different modification of the pegRNA by Jianghuai Liu’s team showed a viral exoribonuclease-resistant RNA motif (xrRNA) further improved editing efficiency [34]. Another modifier of RNA stability was revealed in a CRISPR interference (CRISPRi) screen to identify genes that regulate prime editing efficiency [35]. This screen revealed the protein La, the sole positive regulator of prime editing from this screen, which binds terminal poly-uridine tracts of small RNAs. Fusion of a fragment of the La protein to PEmax, termed PE7, displayed superior editing efficiency compared to the parental system across a range of loci, which was found to be dependent on pegRNAs with a La-accessible 3’ poly-uridine tract.

Another structural issue arises due to the autoinhibitory interaction of the PBS (including the first 3-nt of the RTT) and the spacer sequences as identified by Ponniensevlen and colleagues [36]. Comparing epegRNAs and chemically stabilised pegRNAs in a ribonucleoparticle (RNP) delivery system, the authors were able to alleviate this inhibitory interaction by shortening the classically 13-nt optimised PBS sequence, and subsequently reveal improved PE in primary patient fibroblasts.

### pegRNA modifications for Prime Editing precision

Reverse transcription of the pegRNA scaffold is also a potential by-product of prime editing [37], especially when using highly processive PEs [11] or a prime editing approach that relies on double-stranded DNA breaks (DSBs) [12]. A novel pegRNA modification that can be used with chemically synthesised pegRNAs, the inclusion of a riboabasic spacer (essentially an abasic site) between the RTT and the pegRNA scaffold, was shown to eliminate scaffold integration [14], thereby further improving prime editing precision. However, considering that the inclusion of a riboabasic site in a pegRNA would require chemical synthesis, this strategy would not be amenable to many expression-based delivery strategies such as AAVs.

Further modifications to the RTT of the pegRNA take advantage of sequence homology between the pegRNA and the target genomic DNA. For example, Template Jumping Prime Editing (TJ-PE) was inspired by non-LTR retrotransposons which integrate into the genome via a 3’ PBS that recognises a 3’ flap created by an endonuclease [38]. On the 5’ end of the retrotransposon is a sequence that is reverse complementary to the target DNA. Once reverse transcribed, the newly synthesised sequence is integrated into the target DNA aided by the now complementary 3’ sequence, acting as a second PBS facilitated by a second nicking gRNA. TJ-PE exploits this process by including an additional sequence on the 5’ end of the pegRNA RTT which is reverse complementary to a sequence downstream on the opposite strand. Due to this dual PBS system, TJ-PE is specialised for insertion edits. TJ-PE harnessing PE2 exhibited dramatic improvements in insertion efficiency of a 200-nt sequence into the *PRNP* and *IDS* loci compared to a traditional PE3 system (i.e. a pegRNA lacking a 5’ reverse complementary PBS2). The limits of TJ-PE were tested by employing a TJ-pegRNA to insert an 833-nt splice acceptor/GFP sequence into the *AAVS1* locus where this large construct could be precisely inserted and correctly spliced in 2% of cells. Furthermore, due to the potential for large insertions resulting in a longer and less stable TJ-pegRNA 3’ extension, further modifications to stabilise the RNA are vital. For this reason, a similar split PE system with circularised petRNAs as presented by Liu and colleagues [18] was generated to mitigate issues surrounding the delivery of unstable and lengthy TJ-pegRNAs.

### Paired pegRNA systems

Many groups have focused on the development of dual pegRNA strategies for increased editing efficiencies and longer edit potential. The application of two pegRNAs targeting opposing strands was demonstrated as a method to increase editing efficiency for prime editing in plants [39]. Using a pegRNA pair that targets distal protospacers, the PRIME-del strategy offers the ability to make large deletions (tested up to 10 kb) with just a small RTT for a short resulting insertion sequence [40]. In this work, each pegRNA installs a sequence that is homologous to the other pegRNA followed by a sequence homologous to the downstream genome upstream of the nick, leading to deletion of the sequence between the nicks. Applying pegRNAs with sequences that complement each other at the ends of the RTTs has also been shown as a viable strategy for installing larger sequences [41]. Another iteration of this strategy was presented by Anzalone and colleagues [3], employing pegRNAs that install edits that are homologous to each other but not to the target locus. This twin prime editing (twinPE) approach was able to replace a 90 bp sequence of the *HEK3* locus with an *attB* site at ~80% editing efficiency, or an *attP* site at ~58% editing efficiency. A twinPE system for the installation of *attP* sites in combination with a Bxb1 serine integrase and a 5.6 kb donor sequence with an *attB* site was able to knock-in the donor into the genome at the installed *attP* sites with efficiencies up to 6.8%. It was also shown that twinPE could be employed for genomic inversions by installing *attB* and *attP* sites at loci as distal as 40 kb with efficiencies between 7.7% and 9.6%. Yarnall and colleagues [42] added to this by demonstrating that a modified PE fused to Bxb1 at the RT was able to install *attB* sites and subsequently insert donor DNA in a single delivery. The third generation of this Programmable Addition via Site-specific Targeting Elements (PASTE) strategy showed integration of donor sequences as long as 36 kb via the installed *attB* sites at ~12% efficiency in the *ACTB* locus [42]. Rational engineering as well as evolution of the Bxb1 integrase using PACE and PANCE gave rise to an engineered and evolved Prime editing Assisted Site-Specific Integrase Gene Editing (eePASSIGE) system [43]. Interestingly, untethering the Bxb1 as per PASTE, evolved or not, improved integration efficiency supporting the incompatibility of simultaneous prime editing and Bxb1-mediated integration. In comparison, the split and evolved eePASSIGE system exhibited an average 22% integration efficiency of a 5.6 kb donor plasmid across eight loci compared to 3.8% using the tethered Bxb1 PASTE system.

Another approach investigated the potential of paired pegRNAs to delete large regions followed by a small insertion using a fully active Cas9 nuclease conjugated to an RT. This PE-Cas9-based Deletion and Repair (PEDAR) strategy enabled the precise deletion of 8 kb and 10 kb followed by replacement with 18 bp at the *CDC42* locus with ~18% and ~7% efficiency, respectively [44]. Furthermore, in a murine model of tyrosinemia type I simulated by insertion of a neomycin cassette (1378 bp) in the *Fah* gene, PEDAR was able to restore FAH expression in ~0.76% of hepatocytes one week after administration of a plasmid based PEDAR system. Adding to this, Kweon and colleagues [45] demonstrated that a similar system can be employed to stimulate chromosomal translocations and inversions like those observed in many cancers. This PEn-mediated Translocation and Inversion (PETI) strategy involves design of pegRNAs that target two different loci with RTTs that are homologous with the other locus. Supporting evidence of PETI, the *EML4-ALK* fusion gene which is a feature of many non-small cell lung cancers was simulated in HEK293T cells by applying pegRNAs targeting intronic regions of *EML4* and *ALK* with RTTs homologous to the other locus. These pegRNAs in combination with a PEn induced the expression of the fusion gene transcript which enhanced ALK protein expression compared to a standard Cas9 system, exemplifying the power of PETI to induce chromosomal structural changes.

## Additional Prime Editing modifiers

Whilst the early events of prime editing were well characterised in the initial study [2], a more detailed understanding of the endpoint steps only became clear in later studies. One notable clue to that mechanistic understanding in the original study was the observation that the use of a pegRNA that led to a 3 bp insertion had a generally higher efficiency than pegRNAs that only introduced a single nucleotide substitution. This hinted towards a relationship between the desired edit’s homology with the unedited sequence and prime editing efficiency.

### Mismatch repair modifiers and nucleotide availability

Since the Liu lab’s original paper [2], a litany of studies has contributed towards enhancing the efficiency of prime editing – a key aspect towards shifting this tech to the clinic. These range from targeting cellular processes, sequence optimisation and design, through to spatial reconfiguration of each functional component of the PE system. One of the best examples of this came through a Repair-seq CRISPRi library screen by Liu’s group, indicating that repression of the MMR pathway could enhance prime editing in cell types with robust MMR. Dominant negative variants of MLH1 co-transfected with the PE2 editor alone (a system hence known as PE4) or with the PE2 editor and a nicking guide (PE5), increased both purity (intended vs unintended on-target editing ratio) and efficiency [9]. This study further showed that the use of an RTT sequence with a small number of silent mutations could additionally enhance prime editing, most likely as it is sufficiently different to evade MMR. The installation of these silent substitutions at the site of prime editing increases the mismatches between the edited and unedited strand thereby weakening the heteroduplex intermediate, which may be less amenable to MMR [9]. A protocol paper from the Liu lab describes these options in more detail [37].

Not long after this, Ferreira da Silva and colleagues [46], validated this phenomenon of improved prime editing in conjunction with transient MMR ablation. Colocalisation of the MHL1 protein at Cas9(H840A)-RT binding sites was visibly demonstrated using super-resolution microscopy, thus revealing a potential mechanism in the direct inhibition of intermediate PE structures. In a similar vein, it was shown that inhibition of p53 (via co-transfection of the dominant negative p53DD fragment) was able to increase prime editing two- to three-fold in human pluripotent stem cells [47] (hPSCs). In their study, this increased efficiency appeared to be broadly applicable across substitutions and indels of varying sizes likely due to the inhibition of cell cycle arrest. Temporary manipulation of the endogenous cellular machinery (whether alone, or in a combinatorial manner) is likely to provide significant “easy wins” in increasing prime editing efficiency. Alternatively, supplementing factors that are not readily available may also improve prime editing efficiency. Free deoxynucleotide triphosphates (dNTPs) are required for the DNA synthesis in dividing cells, and therefore are instrumental in the reverse transcription of the edited 3’ flap during prime editing. In the case of quiescent cells such as stem cells, dNTP levels are lower, which reflects mitotic propensity [48]. Indeed, dNTP supplementation increased prime editing efficiency in human stem and progenitor cells (HSPCs), which was further improved by the addition of Vpx [49]. Vpx is a viral protein which targets the SAMHD1 triphosphohydrolase for proteasomal degradation, thereby reducing the catabolism of dNTPs and priming quiescent cells for viral infection and replication. In confirmation of the ability of Vpx to improve prime editing via SAMHD1 degradation, a Q76X Vpx mutant was shown to have no effect on prime editing efficiency in fibroblasts or T cell while a WT Vpx improved different edit types and different loci [48].

Addition of RNA aptamers into the pegRNA to recruit prime editing modifiers has been further explored by incorporating a PP7 aptamer into the pegRNA to recruit a PP7-binding coat protein (PCP)-conjugated 5’-3’ exonuclease [50]. It was postulated that recruitment of a 5’-3’ exonuclease to the prime editing site might resect the 5’ genomic flap created by the nick, thereby favouring the edit on the 3’ flap. Indeed, this Exo-PE strategy displayed superior or comparable editing efficiency compared to other PEs in different edit types, exemplifying the potential for tailoring prime editing by recruiting modifiers using RNA aptamers. There appears to be a common theme in the stabilisation of the pegRNA with others showing optimisation of the pegRNA significantly improving prime editing [51]. In addition to modifications that improve nuclear localisation of the PE as shown by prime editing in animal liver tissue [52], the numerous and at times, incremental but no less important improvements in efficiency, are collectively moving prime editing closer to the clinic.

### Viral-based Prime Editing delivery

Other prime editing modifications have focused on delivery rather than editing efficiency. A lentiviral-derived nanoparticle (LVNP) approach was developed and optimised for the delivery of prime editing machinery [53]. Using LVNPs loaded with PEmax or PEmaxΔRNaseH and an epegRNA targeting the *HEK3* locus for a 3-nt insertion, prime editing efficiencies of 5–6% were observed in HEK293T cells in vitro. Further advancements in viral delivery of prime editing machinery were developed by adapting the engineered virus-like particle (eVLP) systems used for the delivery of base editor components [54]. After many modifications to the prime editing portion of this system including PEmax and epegRNAs with RTTs encoding silent substitutions to evade MMR, an increase in editing efficiency was indeed observed resulting in the v1 PE-eVLP architecture [55]. After modifying the protease-sensitive site in the PEmax RT that is targeted by the M-MLV protease required for eVLP maturation, inserting nuclear export sequences into the Gag structural polyprotein and GGS linkers around the protease cleavage site to improve protease processivity, the eVLP architecture arrived at v2. Finally, modifications to improve epegRNA loading such as including MS2 aptamers to the epegRNA with an MCP incorporated into the Gag-Pol cassette, as well as CC-peptides to improve recruitment of the epegRNA and PEmax to the viral capsid, respectively, the v3 PE-eVLP system was produced. These eVLPs displayed 2–3% prime editing efficiency of a 4-nt insertion into *Dnmt1* 3 weeks after intracerebroventricular injection into neonatal mouse brains, reaching up to 47% when sorting for GFP positivity when coinjected with a lentivirus encoding EGFP. Similarly, subretinal administration of v3 PE-eVLPs designed for the correction of nonsense mutation in the *Rpe65* gene which leads to retinal degeneration in mice. After optimisation, this strategy yielded a 7.2% correction of the mutation in bulk retinal pigment epithelial tissue, correlating to rescue of RPE65 protein expression and subsequent restoration of sight as measure by electroretinogram [55]. These developments emphasise the PE-specific delivery innovations that open the field to in vivo prime editing.

### Manipulating chromatin architecture for Prime Editing

Taking a more endogenous view at the local structure of editing targets rather than delivery, the local chromatin environment of the target locus may influence prime editing efficiency. The potential for chromatin to affect prime editing can be emphasised by the consideration of epigenetics into the aforementioned pegRNA design tool PRIDICT to create ePRIDICT [28]. Song and colleagues were also able to reveal predictions after assessing chromatin state using several epigenetic states [56]. Using a combination of published and in-house experiments, the authors were able to show that the repressive mark H3K9me3, but not H3K27me3 impacted prime editing, with editing close to 0% close to sites occupied by the former. This phenomenon is in line with the more permanent nature of H3K9me3 compared to the transient H3K27me3 deposition. In addition to revealing some predictive occlusion by nucleosome occupancy, the authors importantly acknowledged that local epigenetic marks are sufficient to block editing but not necessary. This could explain the relatively small improvement of histone deacetylase inhibitors (HDACi) in reducing the incidence of small PE-mediated indels, but not single variants [57]. It was hypothesised that the repair mechanisms used to resolve single nucleotide prime edits is different compared to insertions or deletions, and HDACi may therefore have opposing effects by impacting different repair machinery.

The effect of target locus topology and chromatin structure on prime editing efficiency can also be used as a predictive measure for pegRNA design [28]. Another consideration for editing efficiency regarding chromatin state is the locus activity, wherein actively transcribed genes may be more accessible and therefore more easily edited [31]. Rather than modifying the local chromatin structure with HDACi, the application of truncated “dead” single guide RNAs (dsgRNAs) has also been explored to improve prime editing efficiency [58]. PEs fused to small chromatin-modulating peptides similarly displayed the potential for opening local chromatin with subsequent improvements in prime editing efficiency. However, the simple addition of a 14-15nt dsgRNA targeting a protospacer proximal to the site of prime editing significantly improved editing efficiency and opened local chromatin [58] while negating the nuclease activity of the complexed Cas [59]. The application of dsgRNAs in prime editing has been further investigated to improve the editing efficiency of *CFTR* to correct the F508del mutation [31]. It was hypothesised that the chromatin state of a target locus can affect PEs distinctly, as a pegRNA-guided base editor displayed dramatically higher editing efficiency compared to a PE. A dsgRNA proximal to the F508del mutation was applied with PEmax and was found to improve *CFTR* prime editing efficiency by 4.4-fold compared to PEmax alone. Further modifications, such as epegRNAs, silent edits and substituting PEmax for PE6c also improved F508del correction by 20-fold when applied together, but the power of a single dsgRNA is exemplified by further improving the correction of F508del with these other components up to 28-fold compared to PEmax alone without impacting the proportion of indels compared to editing efficiency [31].

## Concluding remarks

A quick search of Pubmed with the term “prime editing” reveals that this technique is mirroring the same growth rate as the early days of base editing. Base editing heralded a seismic shift away from a dependence on double-stranded DNA breaks and has recently entered clinical trials [60] despite a limited selection of possible edits and some concerns regarding off-target effects [61]. In addition, despite the flexibility of prime editing compared to base editing and other strategies, the genotoxic effects of either strategy should be balanced against even against the minimised risk of DSBs compared to nuclease-based approached [61]. Many groups address the potential off-target effects of prime editing by applying strategies such as CIRCLE-seq [62].

Given the enhanced scope of prime editing to correct any single nucleotide polymorphism (SNP), correct multiple mutations with a single pegRNA, and the options to generate a range of more diverse edits, coupled with several studies reporting successful delivery and efficiency of prime editing in a number of in vivo models, including a very recent study in non-human primates [63], the step to the clinic is surely close for prime editing.

One big difference in the development of prime editing when compared to base editing though is the potential need for a deeper level of customisation and optimisation of both the pegRNA and the PE itself. But as with all things, perhaps we should also take steps to ensure that the unquenchable thirst to improve and refine – *nil satis nisi optimum* **–** does not deflect from strategies that could be used to help those with genetic diseases in the near future.

### Note on nomenclature

We have used “Prime editing” in full each time to describe the editing process, and use “PE” as the abbreviation for “Prime editor” when referring to Cas9-RT fusions or Cas9/RT combinations.
